# Privacy-Preserving and Poisoning-Robust Federated Learning for Industrial IoT

**DOI:** 10.3390/s26165297

**Published:** 2026-08-21

**Authors:** Huan Yin, Congwen Chen, Jingyi Zhang, Dian Yu, Shuanggen Liu

**Affiliations:** 1 School of Electrical Engineering, Jiangxi Vocational College of Mechanical and Electrical Technology, Nanchang 330013, China; yinhuan@jxjdxy.edu.cn; 2 School of Cyberspace Security, Xi’an University of Posts and Telecommunications, Xi’an 710121, China; chencongwen@stu.xupt.edu.cn (C.C.); 2402603013@stu.xupt.edu.cn (J.Z.); 2385726127@stu.xupt.edu.cn (D.Y.)

**Keywords:** industrial IoT, anomaly detection, federated learning, privacy protection, robust defense

## Abstract

With the rapid development of Industrial Internet of Things (IIoT), large amounts of sensor data generated by industrial devices and edge nodes have become the basis of intelligent manufacturing applications. Collaborative modeling on these distributed data is important for tasks such as anomaly detection, equipment monitoring, and predictive maintenance. Federated learning offers a practical way to train models without exposing raw sensor data, but it still faces privacy leakage and malicious poisoning attacks. To address these issues, this paper proposes a hierarchical privacy protection and poisoning-robust defense framework for industrial federated learning. Starting from the sensitivity differences among parameters at different model layers, the proposed method designs a hierarchical privacy-budget allocation strategy that enhances protection for sensitive information while minimizing the performance impact of perturbation. Meanwhile, a multi-layer, multi-feature anomaly-detection mechanism is adopted to identify malicious updates by jointly exploiting directional consistency, scale stability, and inter-layer similarity, and majority voting together with update clipping is used to further improve system robustness. Experiments on Fashion-MNIST, MVTec AD, and C-MAPSS demonstrate that the proposed method can effectively suppress global-model degradation under multiple poisoning attacks and achieves a favorable balance among privacy protection strength, robustness, and training efficiency.

## 1. Introduction

### 1.1. Background and Related Work

With the deep integration of next-generation information technologies and advanced manufacturing technologies, intelligent manufacturing is becoming an important direction for promoting high-quality development of the manufacturing industry. In an intelligent manufacturing system, tasks such as production-line visual inspection, equipment-state monitoring, process-parameter analysis, and abnormal-behavior recognition all rely on the continuous accumulation and modeling of large amounts of high-value industrial data [1,2]. However, these data are often stored separately across different factories, production lines, and edge terminals, forming a typical application pattern characterized by data dispersion, scenario heterogeneity, and strong collaboration demands [3]. Because of commercial competition, data-compliance requirements, and intellectual-property protection among enterprises, raw data are usually difficult to aggregate directly on a central server for unified training [4]. This makes cross-entity collaborative modeling without moving data out of the factory a key problem that urgently needs to be solved in intelligent manufacturing.

Federated learning provides a practically feasible technical route for this problem. The paradigm allows each participant to train locally and upload only gradients or parameter updates to the server for aggregation, thereby balancing data utilization efficiency and privacy protection to a certain extent [5]. In intelligent manufacturing scenarios, this mode is particularly suitable for cross-factory defect detection, distributed equipment diagnosis, and multi-line quality assessment tasks because each party hopes to share model capability while refusing to expose raw industrial data [6]. However, federated learning does not mean that the training process is inherently secure. For industrial visual images, equipment operating states, and process parameters, even if raw data do not leave the client, the uploaded model updates may still contain sufficient statistical information for attackers to infer training-sample characteristics, equipment operation patterns, or process distributions. Existing studies have shown that attackers can use gradients or parameter updates to conduct membership inference, attribute inference, and sample-reconstruction attacks, thereby causing industrial-knowledge leakage and business-strategy exposure [7,8,9]. For manufacturing enterprises, such leakage not only affects data privacy but may also destroy process barriers and even undermine the trust foundation in supply-chain collaboration, so the security risk cannot be ignored.

In addition to privacy leakage, federated learning systems in intelligent manufacturing also face model poisoning attacks [10]. In cross-factory collaborative training, the system typically involves multiple edge nodes or enterprise clients, and attackers may control a small number of clients to upload abnormal updates that interfere with the convergence direction of the global model and degrade the performance of defect recognition and industrial prediction tasks. For industrial anomaly detection and quality-control tasks, such attacks may directly cause missed detections, false detections, or incorrect releases, whose consequences are often more severe than those in ordinary classification tasks. In particular, in highly non-IID industrial environments, differences among device types, process flows, and product batches are significant, and even a small number of malicious updates can accumulate over multiple aggregation rounds, continuously damaging the global model [11].

Existing research on privacy and security in federated learning mainly focuses on differential privacy, secure aggregation, homomorphic encryption, multi-party computation, and robust aggregation [12,13]. Differential privacy limits the influence of a single sample on the output by injecting noise into gradients or statistics, but excessive noise can significantly degrade model performance and make it difficult to satisfy the stability and accuracy requirements of industrial inspection [14]. Secure aggregation and homomorphic encryption can hide individual client updates so that the server cannot directly access plaintext information, but this also makes traditional anomaly-detection methods based on gradient distance, directional consistency, or statistical distribution difficult to apply directly [15]. Robust aggregation attempts to identify anomalous clients by exploiting the geometric relationships among updates, but such methods usually assume that the server can observe plaintext updates, which limits their applicability in encrypted environments [12]. It can therefore be seen that, in intelligent manufacturing scenarios, privacy protection and poisoning defense are not independent problems but a systemic contradiction that is mutually constraining and tightly coupled [13].

From an industrial application perspective, intelligent manufacturing imposes stricter requirements on federated learning security mechanisms than general scenarios. On the one hand, manufacturing tasks usually involve high-value production data and process knowledge, and any form of leakage may bring substantial commercial risk. On the other hand, industrial systems also demand higher real-time performance, deployability, and training stability, and overly heavy cryptographic overhead or overly complex defense procedures may hinder practical deployment [1,16,17,18]. Therefore, how to further balance privacy strength, anomaly-detection capability, and system efficiency without exposing raw data has become a core issue in federated-learning security research for intelligent manufacturing [19,20]. Motivated by this need, this paper focuses on cross-factory collaborative modeling in intelligent manufacturing and studies how to achieve high privacy, strong robustness, and practical deployability under the premise that data never leave the factory.

### 1.2. Our Contributions

Addressing the collaborative modeling requirements across factories and production lines in intelligent manufacturing, this paper focuses on problems such as distributed industrial data storage, leakage of update information, and malicious-node interference. Centered on the collaborative optimization of privacy protection and robust training without sharing raw data, we propose a joint defense method that balances privacy, robustness, and deployability. The main contributions are as follows.

First, we propose a hierarchical privacy-budget allocation mechanism. Because model layers differ in semantic expression and sensitivity, differentiated privacy-protection strategies are adopted, and budgets are dynamically allocated according to layer importance to enhance sensitive-information protection while reducing the performance impact of privacy perturbation.

Second, we designed a multi-layer, multi-feature anomaly-detection mechanism. In view of camouflage, scaling, and directional-manipulation attacks that malicious clients may adopt, we jointly used the directional consistency, scale stability, and similarity features of multiple key layers to construct a discrimination criterion, thereby improving the ability to identify complex malicious updates and enhancing robustness in non-IID scenarios.

Third, we validated the effectiveness of the proposed method on intelligent-manufacturing-related tasks. Experiments were conducted in an industrial anomaly-detection scenario, and the method was evaluated in terms of attack–defense effectiveness, privacy strength, and computational overhead. The results show that the proposed method can suppress the performance degradation caused by malicious updates while safeguarding industrial data privacy and achieving a favorable balance among privacy, robustness, and efficiency.

### 1.3. Organization

The remainder of this paper is organized as follows. Section 2 introduces background knowledge on federated learning, fully homomorphic encryption, differential privacy, and similarity-based robust aggregation. Section 3 presents the system design, including the overall architecture, workflow, and threat model. Section 4 introduces the core method of this paper, including hierarchical privacy-budget allocation, encryption-efficient computation, and multi-layer, multi-feature anomaly detection. Section 5 provides the security analysis. Section 6 describes the experimental setup, datasets, attack methods, baseline methods, and evaluation metrics. Finally, Section 7 concludes the paper and discusses future research directions.

## 2. Preliminaries

### 2.1. Federated Learning

**Figure 1:**
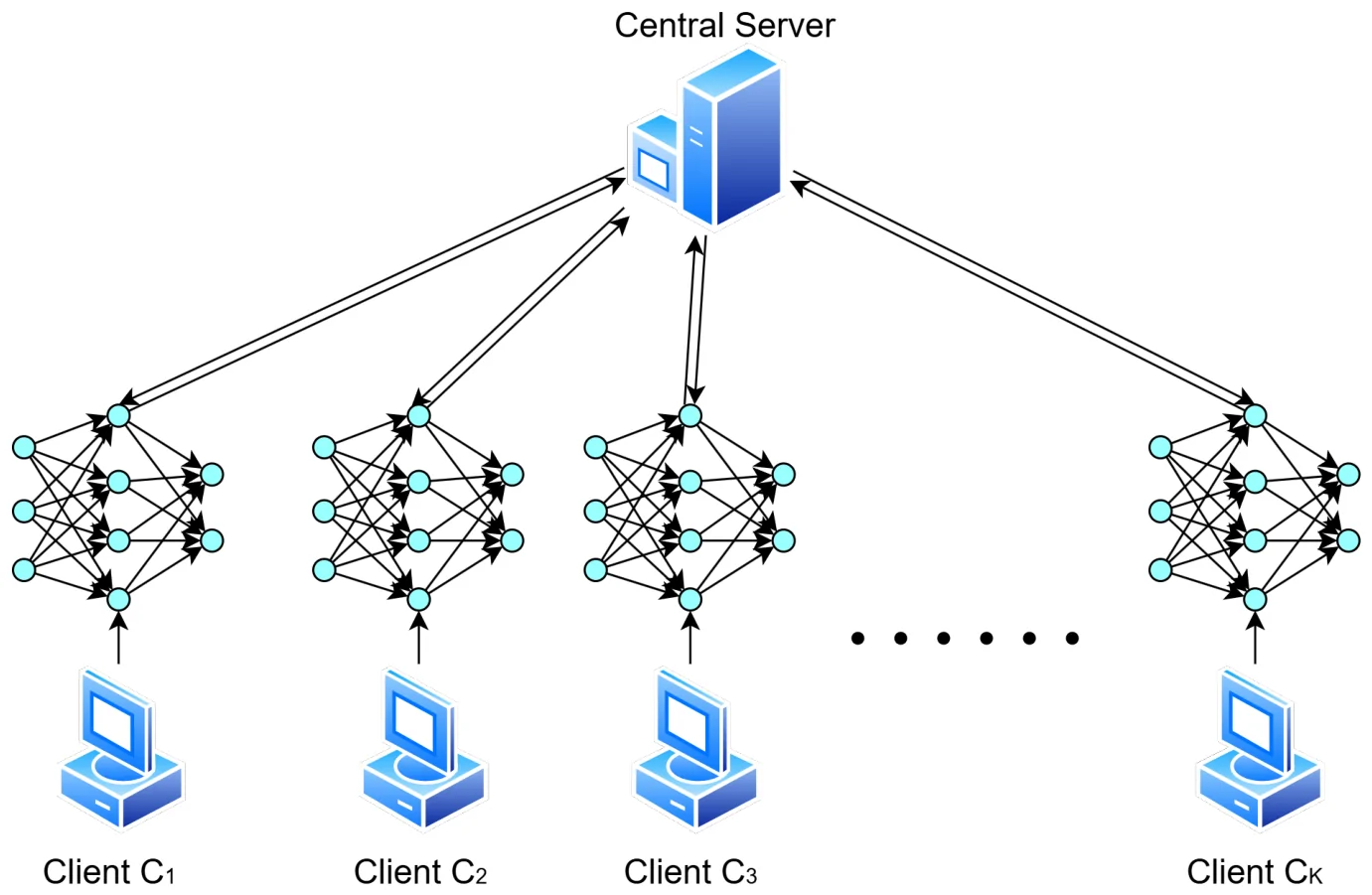
Classical federated learning framework.

As shown in Figure 1, federated learning is a distributed machine-learning paradigm whose core objective is to collaboratively train a global model using local data held by multiple participants without centrally uploading raw data. The paradigm was first systematized by the FedAvg framework and has been widely applied to privacy-preserving collaborative modeling in industrial and intelligent-manufacturing scenarios [5,21]. Unlike centralized learning, federated learning keeps the training data local to the clients and exchanges only model parameters or gradient updates, thereby alleviating privacy risks caused by data centralization to a certain extent. As shown in Figure 1, suppose the system contains *K* clients, denoted by $C_{1},C_{2},\ldots ,C_{K}$, and the *k*-th client holds a local dataset $D_{k}$ with sample size $|D_{k}|$. The basic optimization objective of federated learning can be written as

$$\min_{w}F\left(w\right)=\sum_{k=1}^{K}\frac{|D_{k}|}{\sum_{j=1}^{K}\left|D_{j}\right|}F_{k}\left(w\right),$$

where $F_{k}\left(w\right)$ denotes the empirical loss function defined on client *k*’s local dataset. This shows that the global objective is a weighted sum of local objectives, with weights usually proportional to local data size.

A typical federated-learning process consists of multiple communication rounds. In round *t*, the server first broadcasts the current global model $w^{\left(t\right)}$ to all clients. Each client then performs several steps of gradient descent or stochastic gradient descent on its local data to obtain a local model $w_{k}^{\left(t+1\right)}$. The clients upload their local updates to the server, which updates the global model according to a predefined aggregation rule. The most common aggregation method is FedAvg:

$$w^{\left(t+1\right)}=\sum_{k=1}^{K}\frac{|D_{k}|}{\sum_{j=1}^{K}\left|D_{j}\right|}w_{k}^{\left(t+1\right)}.$$

FedAvg is simple to implement, converges stably, and is suitable for most federated-training scenarios [5]. However, when malicious clients are present, simple averaging directly allows abnormal updates to affect the global model, leading to robustness issues. In addition, in industrial manufacturing scenarios, data from different factories or production lines are often highly heterogeneous, so federated learning must consider communication efficiency, system stability, and cross-device collaboration capability simultaneously [22].

### 2.2. Fully Homomorphic Encryption and the CKKS Scheme

To perform model aggregation and similarity computation without exposing plaintext updates, this paper introduces a fully homomorphic encryption (FHE) mechanism. FHE allows addition and multiplication to be performed directly on ciphertexts while guaranteeing that the decryption result is consistent with the result of performing the same operations on plaintexts. Let the encryption function be Enc(·) and the decryption function be Dec(·). For arbitrary plaintexts and constants, we have

$$\mathrm{Dec}\left(\mathrm{Enc}\left(m_{1}\right)⊕\mathrm{Enc}\left(m_{2}\right)\right)=m_{1}+m_{2}$$

$$\mathrm{Dec}\left(\mathrm{Enc}\left(m_{1}\right)⊗\mathrm{Enc}\left(m_{2}\right)\right)=m_{1}\times m_{2}$$

The core advantage of FHE is that the server can complete necessary computations without seeing plaintext, thereby embedding the privacy-protection mechanism into the federated-learning training flow [23,24]. However, the computational overhead of FHE is usually high, so the operation structure must be carefully designed in practical systems to reduce encryption, decryption, and homomorphic computation costs as much as possible.

The CKKS scheme is a homomorphic encryption scheme designed for approximate real-number computation and is well suited to floating-point model parameters in federated learning. Unlike traditional exact integer homomorphic schemes, CKKS supports approximate representation of real-valued vectors in ciphertexts and enables vectorized encoding and batch operations, which makes it highly suitable for packed encryption of model parameters. Let the real-valued vector *x* be encoded by CKKS into a ciphertext that can be processed uniformly, reducing the number of ciphertext objects and improving computational efficiency [25].

### 2.3. Differential Privacy

Differential privacy is a privacy-preserving mechanism used to quantify the leakage risk of statistical queries [26]. Its basic idea is that when a single sample in the input dataset changes, the output distributions of the algorithm should remain as similar as possible, making it difficult for attackers to determine whether a sample participated in training. A randomized mechanism satisfies (ε,δ)-differential privacy for any pair of adjacent datasets if its output distributions satisfy the corresponding inequality. Here, ε denotes the privacy budget, and δ is a small failure probability. In general, the smaller ε is, the stronger the privacy protection, but the more noise is introduced and the more likely model performance is affected.

One of the most commonly used implementations of differential privacy is the Gaussian mechanism. If the global sensitivity of a function *f* is Δ, then differential privacy can be achieved by adding Gaussian noise:

$$M\left(x\right)=f\left(x\right)+N\left(0,\sigma^{2}\right),\;\sigma \geq \frac{\sqrt{2\ln(1.25/\delta )}\;\Delta }{\varepsilon }.$$

The Gaussian mechanism is attractive because it is simple and naturally compatible with gradient updates and intermediate representations, so it is often used in federated-learning privacy protection. It should be noted that differential privacy does not directly prevent the server from accessing model updates; instead, it reduces the probability that information about a single sample or a single client can be inferred by introducing statistical perturbation. Therefore, differential privacy is usually combined with encryption mechanisms: the former controls leakage at the statistical level, and the latter protects data confidentiality at the communication and computation levels. Only by combining the two can a more complete privacy-protection chain be formed.

### 2.4. Similarity-Based Robust Aggregation

In federated learning, the server usually uses similarity among client updates to determine which updates are more likely to come from benign participants. The intuitive assumption behind similarity-based methods is that when most clients are benign, normal updates tend to be highly consistent in direction, whereas malicious updates are more likely to deviate from the mainstream direction. Based on this idea, cosine similarity can be used to measure directional consistency between a client update and the global model or a reference update.

Let the local update of client *k* in round *t* be $u_{k}^{\left(t\right)}$, and let the reference vector associated with the previous global model be $r^{\left(t\right)}$. Then cosine similarity can be defined as

$$s_{k}^{\left(t\right)}=\frac{\langle u_{k}^{\left(t\right)},r^{\left(t\right)}\rangle }{∥u_{k}^{\left(t\right)}∥\cdot ∥r^{\left(t\right)}∥+\eta }.$$

When $s_{k}^{\left(t\right)}$ is large, the direction of the client update is more consistent with the reference direction and is therefore more likely to be a benign update; otherwise, it may be an anomalous update. Similarity-based methods are intuitive and easy to implement, and they are suitable for server-side anomaly identification. However, such methods usually assume that the server can directly observe plaintext updates, so they inherently conflict with privacy-protection mechanisms. If model updates are fully encrypted, traditional similarity computation cannot be performed directly. Therefore, one of the core goals of the following method is to reconstruct a usable similarity-detection workflow with encryption.

In addition to a single similarity metric, robust aggregation often combines threshold screening, clustering analysis, median estimation, or clipping mechanisms to reduce the impact of anomalous updates on the global model [27]. In general, these methods establish decision criteria based on update direction, magnitude, or historical consistency and remove suspicious clients before aggregation. However, in scenarios where federated learning requires both privacy protection and robustness, how to carry out effective filtering without exposing plaintext updates remains a problem that still needs to be solved.

## 3. System Design

### 3.1. System Architecture

To address problems such as decentralized storage of multi-source industrial data, difficulty in cross-enterprise data sharing, and insufficient security during model training in intelligent manufacturing environments, the architecture proposed in this paper is an improvement based on the basic idea of dual-defense federated-learning design [28]. Without aggregating raw industrial data, the architecture realizes collaborative modeling among industrial nodes through federated learning while combining encrypted computation, privacy perturbation, and anomalous-update detection to improve the security and reliability of the model-training process.

**Figure 2:**
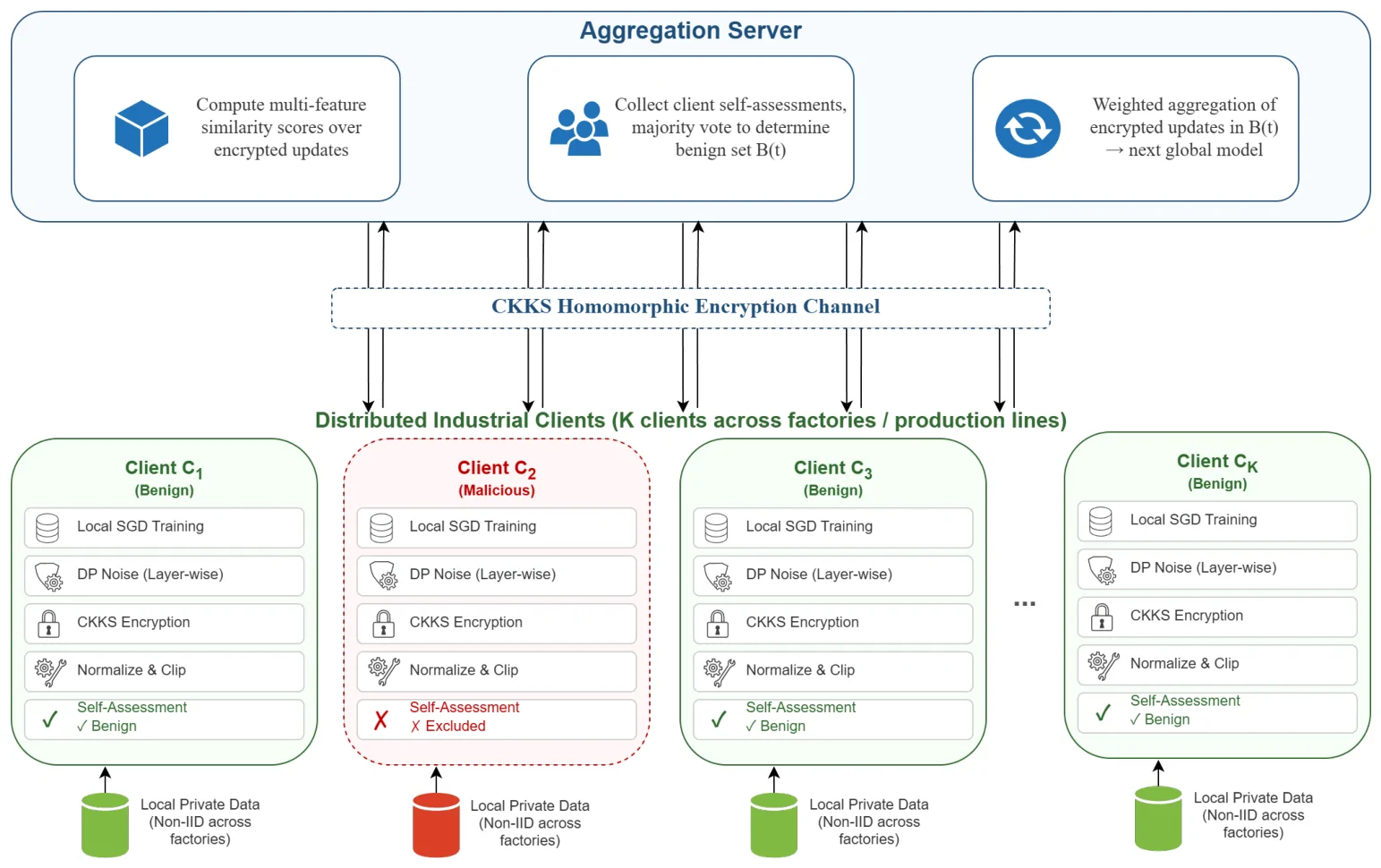
System framework.

As shown in Figure 2, the proposed system mainly consists of industrial clients and an aggregation server. Industrial clients are deployed in different manufacturing enterprises, production lines, or edge-computing nodes. Each client owns independently collected industrial data resources, such as equipment operating-state data, sensor data, and product quality inspection data. Because industrial nodes differ in device type, production environment, and process flow, the local data of each client usually exhibit obvious non-independent and identically distributed (non-IID) characteristics. Given that industrial data typically contain production-process information, equipment operation patterns, and enterprise business characteristics, the present method adopts a federated-learning mode so that each client trains only on local data and shares knowledge across nodes through model-parameter exchange, avoiding the direct flow of raw data among different entities.

The aggregation server is mainly responsible for global-model initialization, model-parameter broadcasting, client-update reception, and final model aggregation. Unlike traditional centralized machine learning, the server does not directly access clients’ raw data and does not obtain unprocessed plaintext model updates; instead, it performs subsequent computations based on encrypted information uploaded by clients. This design reduces the privacy-leakage risk caused by centralized storage of industrial data and meets the data-security requirements of multi-party collaborative training.

As shown in Algorithm 1, the overall training process is conducted as follows. In communication round *t*, the server first broadcasts the encrypted current global model $C_{G}^{\left(t-1\right)}$ to the participating clients. Each client *k* decrypts the received ciphertext locally using its private key to obtain the plaintext global model $w_{G}^{\left(t-1\right)}$ and then performs local training on its dataset $D_{k}$ to derive the locally updated model parameters $w_{k}^{\left(t\right)}$. The corresponding local model update is defined as

$$\Delta_{k}^{\left(t\right)}=w_{k}^{\left(t\right)}-w_{G}^{\left(t-1\right)}.$$

Here, $\Delta_{k}^{\left(t\right)}$ represents the model change generated by client *k* during communication round *t*. To preserve the confidentiality of the complete local update while supporting subsequent anomaly detection, each client encrypts the complete model update using the CKKS homomorphic encryption scheme:

$$C_{k}^{\left(t\right)}={\mathrm{Enc}}_{pk}\left(\Delta_{k}^{\left(t\right)}\right),$$

where $C_{k}^{\left(t\right)}$ denotes the ciphertext of the complete local model update and ${\mathrm{Enc}}_{pk}\left(\cdot \right)$ represents encryption with the public key pk. The complete model update therefore remains encrypted throughout the communication and aggregation process, and the aggregation server does not obtain its plaintext form.
**Algorithm 1** System workflow**Require:** Client set $C=C_{1},C_{2},\ldots ,C_{K}$, initial global model $C_{G}^{\left(0\right)}$, total communication rounds *T***Ensure:** Final global model $w_{G}^{\left(T\right)}$  1:Initialize the global model $C_{G}^{\left(0\right)}$  2:Initialize the cryptographic context and key pair  3:**for** t=1 to *T* **do**  4:   The server broadcasts $C_{G}^{\left(t-1\right)}$ to all participating clients  5:   **for all** clients $C_{k}$ in parallel **do**  6:      $C_{k}$ receives $C_{G}^{\left(t-1\right)}$ and decrypt it using sk to obtain $w_{G}^{\left(t-1\right)}$  7:      $C_{k}$ trains locally and obtains $w_{k}^{\left(t\right)}$  8:      $C_{k}$ computes the local update $\Delta_{k}^{\left(t\right)}=w_{k}^{\left(t\right)}-w_{G}^{\left(t-1\right)}$  9:      $C_{k}$ performs clipping and normalization on the key layers, and injects layer-wise differential privacy noise10:      $C_{k}$ encrypts the processed update using CKKS and obtains $C_{k}^{\left(t\right)}$11:      $C_{k}$ uploads the encrypted update to the server12:   **end for**13:   The server receives all ciphertext updates14:   The server computes multi-feature anomaly scores among key layers in the ciphertext domain15:   The server sends the encrypted scoring results back to each client16:   **for all** clients $C_{k}$ in parallel **do**17:      Decrypt the similarity score18:      Generate the benign candidate set $B_{k}^{\left(t\right)}$ according to the thresholding rule19:      Send $B_{k}^{\left(t\right)}$ back to the server20:   **end for**21:   The server performs majority voting over all $B_{k}^{\left(t\right)}$ and obtains the final benign set $B^{\left(t\right)}$22:   Compute aggregation weights $\lambda_{k}^{\left(t\right)}$ according to data proportions23:   The server performs weighted homomorphic aggregation over $C_{k}^{\left(t\right)},k\in B^{\left(t\right)}$, obtaining encrypted global model $C_{G}^{\left(t\right)}$24:**end for**25:**return** $w_{G}^{\left(T\right)}$

For anomaly detection, the server extracts the ciphertext representations corresponding to the predefined key layers from $C_{k}^{\left(t\right)}$ without performing decryption. These key-layer representations are subsequently used to construct the features required for multi-layer and multi-feature anomaly detection. Specifically, the key-layer representations are clipped and normalized to reduce the influence of abnormal update magnitudes, while the hierarchical differential-privacy mechanism is incorporated into the detection process according to the sensitivity of different layers. The resulting representations remain protected in the ciphertext domain during the subsequent similarity computation. Therefore, the anomaly-detection process operates on encrypted key-layer representations, whereas the original complete-model ciphertext $C_{k}^{\left(t\right)}$ is retained for the final model aggregation.

After receiving the encrypted updates from all participating clients, the server performs the predefined anomaly-detection operations on the extracted key-layer ciphertexts and obtains the corresponding encrypted detection results. The detection results are then transmitted to the participating clients, which locally decrypt the results and generate candidate benign sets according to the prescribed screening rules. To reduce the influence of individual client decisions on the final filtering result, a majority-voting mechanism is employed to determine the final trusted client set $B^{\left(t\right)}$.

After the trusted client set is determined, the server selects the complete-model ciphertexts corresponding to the clients in $B^{\left(t\right)}$ and performs sample-size-weighted homomorphic aggregation directly in the ciphertext domain:

$$C_{G}^{\left(t+1\right)}=\sum_{k\in B^{\left(t\right)}}\frac{|D_{k}|}{\sum_{j\in B^{\left(t\right)}}\left|D_{j}\right|}⊗C_{k}^{\left(t\right)},$$

where $C_{G}^{\left(t+1\right)}$ denotes the encrypted global model obtained after secure aggregation, and ⊗ represents the ciphertext-domain multiplication by a plaintext aggregation weight. Since the aggregation is performed directly on encrypted complete-model updates, the server does not need to recover any individual plaintext update or the plaintext aggregated model.

The encrypted global model $C_{G}^{\left(t\right)}$ is subsequently broadcast to the participating clients. Each client uses its private key to decrypt the aggregated ciphertext locally and obtains the next-round global model $w^{\left(t+1\right)}$. In this manner, the proposed framework integrates ciphertext-domain anomaly detection and homomorphic model aggregation into a unified privacy-preserving training process while preventing the aggregation server from directly accessing individual plaintext model updates or the plaintext global model.

### 3.2. Threat Model

This paper assumes a semi-honest aggregation server and a set of potentially malicious industrial clients. The server follows the prescribed protocol for model broadcasting, ciphertext computation, anomaly detection, and aggregation but may attempt to infer client-side information from observable communication outputs or detection feedback. Malicious clients may deviate from the prescribed training procedure and upload manipulated model updates to impair global-model convergence or prediction performance.

Let *K* denote the total number of participating clients, *m* denote the number of malicious clients, and f=m/K denote the malicious-client proportion. The default assumption is f<0.5, or equivalently m<K/2, which ensures that benign clients remain the numerical majority required by the subsequent majority-voting mechanism. Unless otherwise stated, the experiments use f=0.2, and the robustness analysis further considers f∈{0.1,0.2,0.3,0.4}.

At the privacy level, adversaries may attempt to infer local training characteristics, device-state information, or process-related features from observable model exchanges and anomaly-detection feedback. At the integrity level, malicious clients may employ statistically camouflaged, magnitude-based, or direction-manipulation updates, including ALIE, Gaussian, IPM, Random Model, scaling, and Sign Flip attacks. The present threat model focuses on untargeted model-poisoning attacks under a malicious-client minority assumption.

## 4. Our Approach

### 4.1. Hierarchical Privacy Budget Allocation

In industrial federated learning, different network layers carry significantly different levels of information sensitivity. In general, shallow-layer parameters mainly reflect general features such as edges and local shapes, while deep-layer parameters are more likely to encode task-specific information related to defect discrimination, equipment-state recognition, and process patterns and thus carry higher privacy-leakage risk. If the same privacy perturbation strength is applied to all layers, shallow layers may be perturbed too strongly, while deep layers may be insufficiently protected, which would affect both model performance and privacy security. To this end, this paper proposes a hierarchical privacy-budget allocation mechanism that assigns different differential-privacy budgets to different layers according to sensitivity differences.

Let the set of key layers involved in protection be $L=\{l_{1},l_{2},\ldots ,l_{R}\}$, and let the total privacy budget be ε. To make the hierarchical budget allocation reproducible, the sensitivity $S_{l}$ of layer *l* is estimated from the normalized magnitude of the layer’s update representation.

Specifically, for client *k* at communication round *t*, the per-client layer-wise sensitivity is first measured by the normalized $L_{2}$-norm of the corresponding update:

$$s_{k,l}^{\left(t\right)}=\frac{{∥u_{k,l}^{\left(t\right)}∥}_{2}}{\sum_{j\in L}{∥u_{k,j}^{\left(t\right)}∥}_{2}+\eta },$$

where η is a numerical stability term. The overall sensitivity of layer *l* at round *t* is then obtained by averaging the normalized layer-wise statistics across all participating clients:

$$S_{l}^{\left(t\right)}=\frac{1}{|C_{t}|}\sum_{k\in C_{t}}s_{k,l}^{\left(t\right)},$$

where $C_{t}$ denotes the set of participating clients in round *t*. To avoid excessive fluctuation caused by individual client updates, the resulting sensitivity values are further normalized before being used in the budget allocation rule. In addition, sensitivity estimation is performed on clipped layer representations, which prevents a single client from arbitrarily inflating the estimated sensitivity via an unbounded update magnitude.

With the layer sensitivity $S_{l}$ (i.e., $S_{l}^{\left(t\right)}$ for the current round) defined, the privacy budget allocated to layer *l* is given by

$$\varepsilon_{l}=\varepsilon \cdot \frac{{\left(S_{l}+\eta \right)}^{-\beta }}{\sum_{j\in L}{\left(S_{j}+\eta \right)}^{-\beta }}.$$

Here β is a budget-allocation control parameter that adjusts the skew of the inter-layer budget distribution. This allocation rule follows the principle that the higher the layer sensitivity, the smaller the allocated privacy budget and the stronger the injected noise, yielding stronger privacy protection; conversely, layers with lower sensitivity are assigned a relatively larger budget to minimize the degradation of the model’s expressive capacity.

For differential-privacy implementation, the present method adopts the Gaussian mechanism to perturb the key-layer representations. For the representation $h_{l}$ clipped and normalized at layer *l*, the perturbed result is

$${\tilde{h}}_{l}=h_{l}+N\left(0,\sigma_{l}^{2}\right),$$

where the noise standard deviation $\sigma_{l}$ can be given by

$$\sigma_{l}\geq \frac{\sqrt{2\ln(1.25/\delta_{l})}\;S_{l}}{\varepsilon_{l}},$$

and $\delta_{l}$ is the failure probability parameter corresponding to layer *l*. With this design, the system can implement differentiated protection according to layer importance and avoid the performance loss caused by using the same perturbation strength for all layers.

It should be emphasized that the differential privacy used in this paper does not directly act on the final aggregation path. Instead, it is mainly used to protect intermediate representations during the anomaly-detection stage. Specifically, after local training, the client first clips and normalizes the key-layer representations used for similarity detection, then injects Gaussian noise according to the hierarchical budgets, and finally uses the perturbed representations for subsequent ciphertext similarity computation and feedback filtering. The purpose of this design is to reduce the risk that the server or malicious clients can infer local update characteristics from similarity feedback, ranking results, or changes in candidate sets while not significantly weakening the final aggregation effect.

In multi-round federated training, differential-privacy loss accumulates across communication rounds and protected layers. Therefore, this paper further combines privacy accounting to track the overall privacy cost in a unified way. Let the privacy consumption of layer *l* in round *t* be $\varepsilon_{l}^{\left(t\right)}$. Under the basic sequential composition theorem, the cumulative privacy-consumption indicator over the entire training process can be expressed as

$$\varepsilon_{\mathrm{sum}}=\sum_{t=1}^{T}\sum_{l\in L}\varepsilon_{l}^{\left(t\right)}.$$

This simple sequential composition provides a conservative upper bound on cumulative privacy consumption for basic composition. However, this bound may be substantially looser than an accountant based on Rényi differential privacy or other advanced composition techniques. Therefore, in the present study, $\varepsilon_{\mathrm{sum}}$ is used as a privacy-consumption indicator rather than as a tight end-to-end privacy guarantee. A tighter privacy accountant and a complete end-to-end privacy certificate are left for future work.

Through this design, differential privacy is extended from a single uniform perturbation to a differentiated protection strategy oriented toward layer sensitivity, which can both strengthen the privacy security of key representations in industrial federated learning and preserve training stability and final model performance as much as possible.

It should be clarified that the differential-privacy mechanism considered in this paper is applied to the client-side intermediate representations used for encrypted anomaly detection rather than directly to the final aggregated model. Accordingly, the privacy mechanism is intended to reduce inference risk from intermediate update characteristics and detection feedback. It should not be interpreted as a complete client-level or central differential-privacy guarantee for the final global model. The privacy analysis in this work therefore focuses on representation-level protection during the anomaly-detection stage.

### 4.2. Multi-Layer, Multi-Feature Anomaly Detection

In federated learning for intelligent manufacturing, the detection of model-poisoning attacks faces two core challenges. First, the inherent process noise and non-IID distribution of industrial data cause substantial statistical fluctuations in the updates of normal clients, which makes anomalous signals from malicious updates easily concealed within legitimate variation and difficult to distinguish. Second, attackers in industrial scenarios can launch highly stealthy and carefully disguised poisoning attacks by crafting malicious updates that closely match the distribution of normal updates, which further raises the difficulty of accurate attack identification. To address these issues, this paper proposes a multi-layer, multi-feature anomaly-detection mechanism. It constructs a composite anomaly score from two complementary dimensions—directional consistency and scale stability—across multiple key layers and filters out malicious updates based on this score.

Let the update representation of client *k* on key layer *l* in round *t* be $u_{k,l}^{\left(t\right)}$, and let the corresponding reference vector be $r_{l}^{\left(t\right)}$. Then the directional similarity of this layer can be defined as

$$s_{k,l}^{\left(t\right)}=\frac{\langle u_{k,l}^{\left(t\right)},r_{l}^{\left(t\right)}\rangle }{∥u_{k,l}^{\left(t\right)}∥\cdot ∥r_{l}^{\left(t\right)}∥+\eta }.$$

Based on layer-wise similarities, we constructed a directional-consistency score to characterize whether the directions across multiple key layers deviate from the mainstream direction synchronously. If a client remains consistent with the mainstream update direction in most key layers, it is more likely to be a benign node; if it only masquerades in some layers while deviating obviously in others, the directional-consistency score will drop significantly, improving the detection capability for selective and targeted poisoning. The directional-consistency term is defined as

$$D_{k}^{\left(t\right)}=1-\frac{1}{R}\sum_{l\in L}s_{k,l}^{\left(t\right)},$$

and the larger its value, the more pronounced the deviation of the client update from the group mainstream direction.

Complementing directional consistency, the scale-stability score is used to detect attacks with normal direction but abnormal magnitude, such as scaling attacks. We consider both the global relative norm deviation and the historical self-consistency deviation. The former reflects the abnormality of the current-round update relative to the group statistics, while the latter reflects its deviation from its own historical trajectory. Let the overall update norm of client *k* in round *t* be $∥u_{k}^{\left(t\right)}∥$, and let the median of group norms be $\mathrm{Med}(∥u^{\left(t\right)}∥)$. Then the scale deviation is

$$R_{k}^{\left(t\right)}=\frac{\left|∥u_{k}^{\left(t\right)}∥-\mathrm{Med}(∥u^{\left(t\right)}∥)\right|}{\mathrm{Med}(∥u^{\left(t\right)}∥)+\eta }.$$

If the historical trajectory is also considered, an inter-round norm-difference term can be introduced:

$$H_{k}^{\left(t\right)}=\frac{\left|∥u_{k}^{\left(t\right)}∥-∥u_{k}^{\left(t-1\right)}∥\right|}{∥u_{k}^{\left(t-1\right)}∥+\eta }.$$

Taking the larger of the two terms allows the method to cover both isolated large-magnitude attacks and progressive magnitude manipulation. Therefore, the scale-stability term is defined as

$$S_{k}^{\left(t\right)}=\max\left(R_{k}^{\left(t\right)},H_{k}^{\left(t\right)}\right).$$

The final composite anomaly score of client *k* in round *t* is obtained by weighting the directional-consistency term and the scale-stability term:

$$A_{k}^{\left(t\right)}=\alpha D_{k}^{\left(t\right)}+\left(1-\alpha \right)S_{k}^{\left(t\right)},$$

where α∈(0,1) balances the two types of features. Considering that update magnitude naturally fluctuates in industrial non-IID settings, whereas direction better reflects whether a client is training along the global optimization objective, the present method adopts a setting in which directional consistency receives slightly greater weight, i.e., α is set to a relatively larger value.

After obtaining the composite anomaly scores of all clients, the system first applies dynamic threshold screening based on the interquartile range to identify suspicious nodes. Let the score set of all clients in the current round be $\left\{A_{1}^{\left(t\right)},A_{2}^{\left(t\right)},\ldots ,A_{K}^{\left(t\right)}\right\}$, with median $Q_{2}^{\left(t\right)}$, upper quartile $Q_{3}^{\left(t\right)}$, and lower quartile $Q_{1}^{\left(t\right)}$. Then the dynamic threshold is defined as

$$\tau^{\left(t\right)}=Q_{3}^{\left(t\right)}+1.5\left(Q_{3}^{\left(t\right)}-Q_{1}^{\left(t\right)}\right).$$

When $A_{k}^{\left(t\right)}\leq \tau^{\left(t\right)}$, client *k* is treated as a benign candidate; otherwise it is marked as suspicious. This mechanism can adapt to changes in the training stage and maintain the statistical dominance of the benign set when the number of malicious clients is less than half.

On this basis, the server feeds back the similarity results computed in ciphertext space to the clients, who decrypt them and perform local judgments before returning candidate sets. Let client *k*’s benign judgment on the *i*-th client be $v_{k,i}^{\left(t\right)}\in \left\{0,1\right\}$, then the final vote count of client *i* is

$$V_{i}^{\left(t\right)}=\sum_{k=1}^{K}v_{k,i}^{\left(t\right)}.$$

When $V_{i}^{\left(t\right)}\geq K/2$, client *i* is included in the final benign set $B^{\left(t\right)}$. This majority-voting mechanism exploits the numerical advantage of benign clients to statistically suppress malicious clients, thereby reducing the influence of feedback-manipulation attacks on the screening result.

Finally, norm clipping is applied to the updates in the benign set to limit the influence of abnormal large-magnitude updates on the global model. If client *k*’s update is $u_{k}^{\left(t\right)}$, then the clipped update is

$${\bar{u}}_{k}^{\left(t\right)}=u_{k}^{\left(t\right)}\cdot \min\left(1,\frac{C}{∥u_{k}^{\left(t\right)}∥+\eta }\right),$$

where *C* is the clipping threshold. This operation effectively constrains the effect of abnormal large-magnitude updates such as scaling attacks, making it difficult for a single malicious client to dominate the aggregation direction. Therefore, the proposed multi-layer, multi-feature anomaly-detection mechanism forms a joint defense chain composed of directional consistency, scale stability, dynamic thresholding, majority voting, and update clipping, enabling effective identification and suppression of complex poisoning behaviors without relying on plaintext updates and providing trusted inputs for subsequent secure aggregation.

## 5. Security Analysis

### 5.1. Confidentiality Analysis

The proposed scheme first protects local updates uploaded by clients through CKKS homomorphic encryption. Let the local update uploaded by client *k* in round *t* be $\Delta_{k}^{\left(t\right)}$. After clipping, normalization, and hierarchical differential-privacy perturbation, the result is ${\tilde{\Delta }}_{k}^{\left(t\right)}$, whose ciphertext representation is

$$C_{k}^{\left(t\right)}={\mathrm{Enc}}_{pk}\;\left({\tilde{\Delta }}_{k}^{\left(t\right)}\right).$$

Under the semantic-security assumption of CKKS, the server can only observe the ciphertext $C_{k}^{\left(t\right)}$ and cannot effectively recover the corresponding plaintext update ${\tilde{\Delta }}_{k}^{\left(t\right)}$. In other words, even if the server knows the entire training interaction process, it is difficult to infer the client’s local parameters, process features, or equipment-state information from ciphertext observations. This property provides a basic cryptographic confidentiality boundary for communication and intermediate computation in industrial federated learning.

Furthermore, in the anomaly-detection stage, only encrypted computation on key-layer representations is performed, and the differential-privacy noise is not directly applied to the final aggregation main path, so the privacy-protection mechanism does not unnecessarily damage the main training process of the global model. Although the server can perform predefined homomorphic operations in the ciphertext domain and generate similarity-related feedback, it still cannot access plaintext updates, so it is difficult to reconstruct fine-grained update structures from one or multiple rounds of communication. Therefore, the proposed scheme can maintain strong confidentiality while preserving collaborative-computation capability.

### 5.2. Inference Suppression Analysis of Hierarchical Privacy Budgets

To reduce the inference risk caused by similarity feedback and candidate-set changes, this paper introduces a hierarchical privacy-budget allocation mechanism in the anomaly-detection stage. Let the key-layer set be L and the privacy budget allocated to layer *l* be $\varepsilon_{l}$, with noise satisfying the Gaussian mechanism:

$${\tilde{h}}_{l}=h_{l}+N\left(0,\sigma_{l}^{2}\right),\;\sigma_{l}\geq \frac{\sqrt{2\ln(1.25/\delta_{l})}\;S_{l}}{\varepsilon_{l}},$$

where $h_{l}$ denotes the clipped-and-normalized representation of layer *l*, $S_{l}$ is its sensitivity, and $\delta_{l}$ is the failure-probability parameter. Since each layer satisfies local differential-privacy constraints, an attacker can only obtain noisy statistical information even after observing the final similarity feedback, and cannot stably recover the fine-grained update characteristics of a client on a single layer.

Let the privacy consumption of layer *l* in round *t* be $\varepsilon_{l}^{\left(t\right)}$. Then the total privacy loss over the full training process can be accumulated by the composition theorem as

$$\varepsilon_{\mathrm{total}}=\sum_{t=1}^{T}\sum_{l\in L}\varepsilon_{l}^{\left(t\right)}.$$

This shows that the long-term privacy cost of the system is traceable and controllable. Compared with directly using a uniform noise level over the whole model, the hierarchical-budget mechanism can apply stronger protection to highly sensitive layers while preserving higher utility for less sensitive layers, thereby achieving a better balance between privacy strength and training performance.

In addition, the feedback information in this paper follows the principle of minimal return, meaning that the server only returns the minimum necessary statistics related to anomaly discrimination rather than exposing the complete intermediate-update structure. Combined with hierarchical noise protection, even if an attacker attempts to infer the states of other clients by observing candidate sets or similarity rankings over multiple rounds, the information available for exploitation is significantly reduced, weakening feedback-based privacy inference.

### 5.3. Poisoning Suppression Analysis

From the perspective of robustness, the main advantages of the proposed scheme lie in four stages: multi-layer, multi-feature anomaly detection; dynamic-threshold screening; majority voting; and update clipping. Let client *k*’s directional-consistency term and scale-stability term in round *t* be $D_{k}^{\left(t\right)}$ and $S_{k}^{\left(t\right)}$, respectively. The composite anomaly score is defined as

$$A_{k}^{\left(t\right)}=\alpha D_{k}^{\left(t\right)}+\left(1-\alpha \right)S_{k}^{\left(t\right)}.$$

Compared with detection methods that rely only on a single-layer similarity metric, multi-layer joint scoring can increase the difficulty of disguising malicious updates. If an attacker tries to evade detection, it must not only mimic the normal update direction on one layer but also maintain directional consistency and magnitude stability across multiple key layers, which greatly compresses the feasible attack space. Therefore, at the same attack strength, multi-layer detection usually has higher identification capability for scaling attacks, directional-manipulation attacks, and Gaussian camouflage attacks.

During screening, the system constructs a dynamic threshold $\tau^{\left(t\right)}$ based on the interquartile range and treats clients satisfying $A_{k}^{\left(t\right)}\leq \tau^{\left(t\right)}$ as benign candidates. Let the benign vote count obtained by client *i* in round *t* be

$$V_{i}^{\left(t\right)}=\sum_{k=1}^{K}v_{k,i}^{\left(t\right)},$$

where $v_{k,i}^{\left(t\right)}\in \left\{0,1\right\}$ denotes client *k*’s binary judgment on client *i*. Client *i* is included in the final benign set $B^{\left(t\right)}$ when $V_{i}^{\left(t\right)}\geq K/2$.

Because the benign clients are assumed to constitute a numerical majority, majority voting increases the likelihood that consistent benign judgments dominate the final client-selection decision. However, this mechanism does not by itself guarantee correct classification of every client. Its effectiveness depends on the consistency of benign-client judgments, the level of differential privacy perturbation, the approximation error introduced by CKKS computation, and the degree of non-IID data heterogeneity. Therefore, majority voting is treated as a collaborative filtering mechanism rather than a formal Byzantine-robustness guarantee.

Before final aggregation, the model further applies norm clipping to the updates in the benign set. If client *k*’s update is $u_{k}^{\left(t\right)}$, then the clipped update is

$${\bar{u}}_{k}^{\left(t\right)}=u_{k}^{\left(t\right)}\cdot \min\left(1,\frac{C}{∥u_{k}^{\left(t\right)}∥+\eta }\right),$$

where *C* is the clipping threshold and η is a numerical stability term. This operation effectively limits the marginal influence of abnormal large-magnitude updates on the global model, especially for malicious behaviors such as scaling attacks that aim to amplify local update magnitudes. Even if the attacker controls one or a few clients, their contribution is restricted to a bounded range and thus cannot dominate the aggregation direction of the global model.

### 5.4. Security Scope and Limitations

The security properties of the proposed framework arise from three complementary mechanisms with different protection objectives. First, CKKS provides cryptographic confidentiality for the encrypted representations and prevents the aggregation server from directly observing their plaintext values with the assumed cryptographic security setting. Second, differential privacy reduces the inference risk associated with the intermediate representations and anomaly-detection feedback by perturbing the protected layer representations. Third, the multi-layer anomaly-detection and voting mechanisms aim to improve resilience against malicious model updates and therefore address integrity rather than confidentiality.

These mechanisms should not be interpreted as providing a universal security guarantee against all possible adversarial behaviors. In particular, the current protocol does not formally analyze collusion between multiple malicious clients and the server, replay or rollback attacks, malformed-ciphertext attacks, adaptive attackers that explicitly optimize their updates against the screening rule, or leakage arising from long-term observation of global-model outputs. In addition, majority voting relies on the existence of a benign-client majority and therefore does not guarantee robustness when malicious clients become the majority. These scenarios are considered important directions for further investigation.

## 6. Experimental Evaluation

### 6.1. Experimental Setup

The experiments were designed to systematically verify the comprehensive performance of the proposed method in terms of privacy protection, poisoning defense, and training efficiency. The experiments were conducted on a computer equipped with an NVIDIA RTX 4070 GPU, an Intel Core i7-14700HX CPU, and 32 GB of memory, and the overall experimental framework was implemented based on PyTorch 2.7.1. To approximate the data-distribution characteristics of real federated-learning environments as closely as possible, all experiments were performed with non-IID settings with tailored partitioning strategies for each dataset. The full experimental pipeline covers data preprocessing, client partitioning, local training, malicious-update injection, encrypted detection, and global model aggregation. Three public datasets were adopted: Fashion-MNIST, MVTec AD, and C-MAPSS. Fashion-MNIST is used to verify effectiveness on standard image-classification tasks, MVTec AD is employed to evaluate defense capability in industrial anomaly-detection scenarios, and C-MAPSS is utilized to assess applicability to remaining-useful-life prediction for industrial equipment.

For client partitioning, specific non-IID allocation rules were designed for each dataset to simulate the data heterogeneity commonly observed among terminal devices or industrial nodes. For Fashion-MNIST, non-IID client partitions were generated using a Dirichlet-based label-distribution strategy, where each client is assigned a distinct class proportion to mimic heterogeneous production environments. The same fixed data partition was applied to all compared defense methods in each experimental run to guarantee fair comparison. For C-MAPSS, temporal samples were distributed across clients according to the engine/subset partition, which preserved client-specific differences in operating conditions. For MVTec AD, a category-based federated allocation strategy was adopted: different product categories were randomly assigned to different clients, and each client only held normal samples from a single category. This setting simulated a realistic federated scenario where multiple factories collaborate on quality inspection while raw data cannot be shared, which better reflects practical industrial challenges such as data silos, scenario discrepancy, and imbalanced sample distribution.

For Fashion-MNIST, the classification model was built upon a lightweight CNN architecture and trained with SGD at a learning rate of 0.01 and a momentum of 0.9, with a batch size of 64 and 5 local epochs in each communication round. For MVTec AD, a pretrained ResNet-18 backbone was utilized as the feature extractor. All input images were resized to 224×224 and normalized following ImageNet statistics. Only defect-free normal samples were used for local training, while anomalous samples were included in the global evaluation phase. The image-level anomaly score was calculated based on the distance between the extracted deep feature embedding and the learned normal feature prototype. For C-MAPSS, experiments were performed on the FD001 subset. The raw sensor sequences were segmented into samples via a sliding window of length W=50 with a stride of 1. The remaining-useful-life target was truncated at 130 cycles using piecewise-linear clipping, and 21 sensor channels were retained after preprocessing. The constructed time-series samples were standardized with statistics computed from the training set. The regression model adopted a CmapssLSTM architecture, optimized by SGD with a momentum of 0.9, a learning rate of 0.001, a batch size of 64, and 10 local epochs per communication round. Mean squared error served as the training loss, and root mean squared error was taken as the primary evaluation metric.

**Figure 3:**
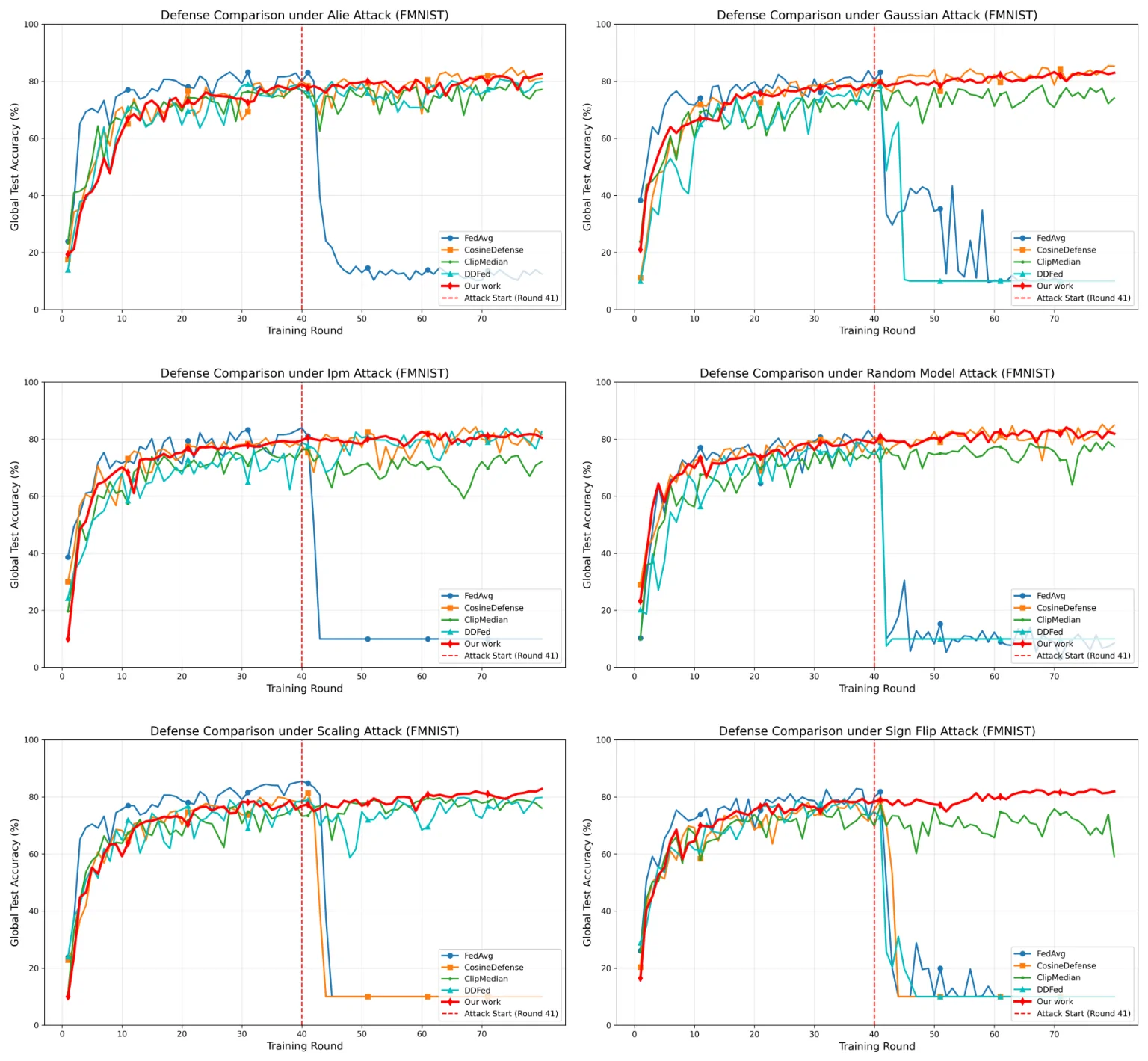
Test accuracy curves of different defense methods in various attack scenarios on the Fashion-MNIST dataset.

**Figure 4:**
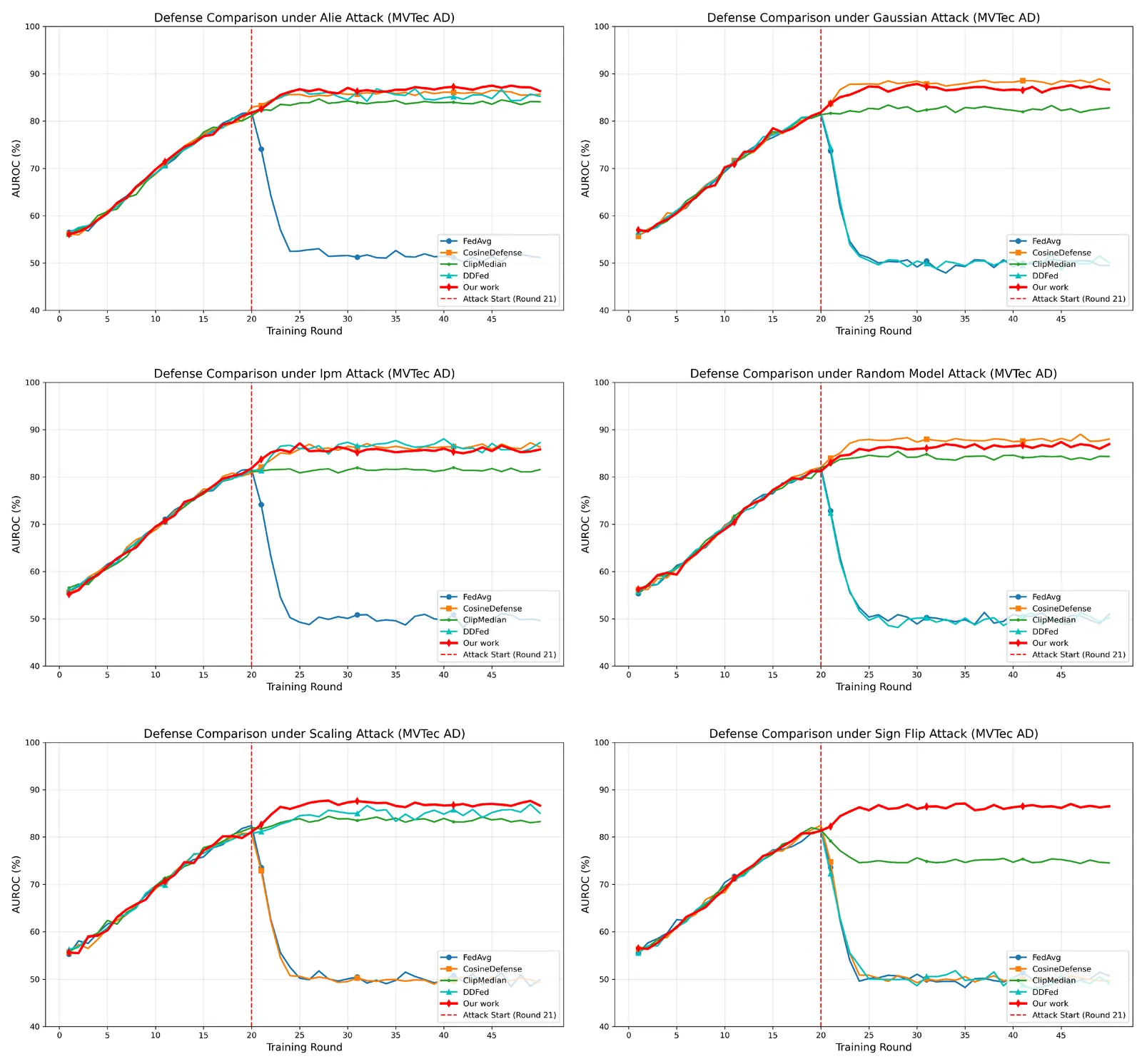
AUROC curves of different defense methods in various attack scenarios on the MVTec AD dataset.

**Figure 5:**
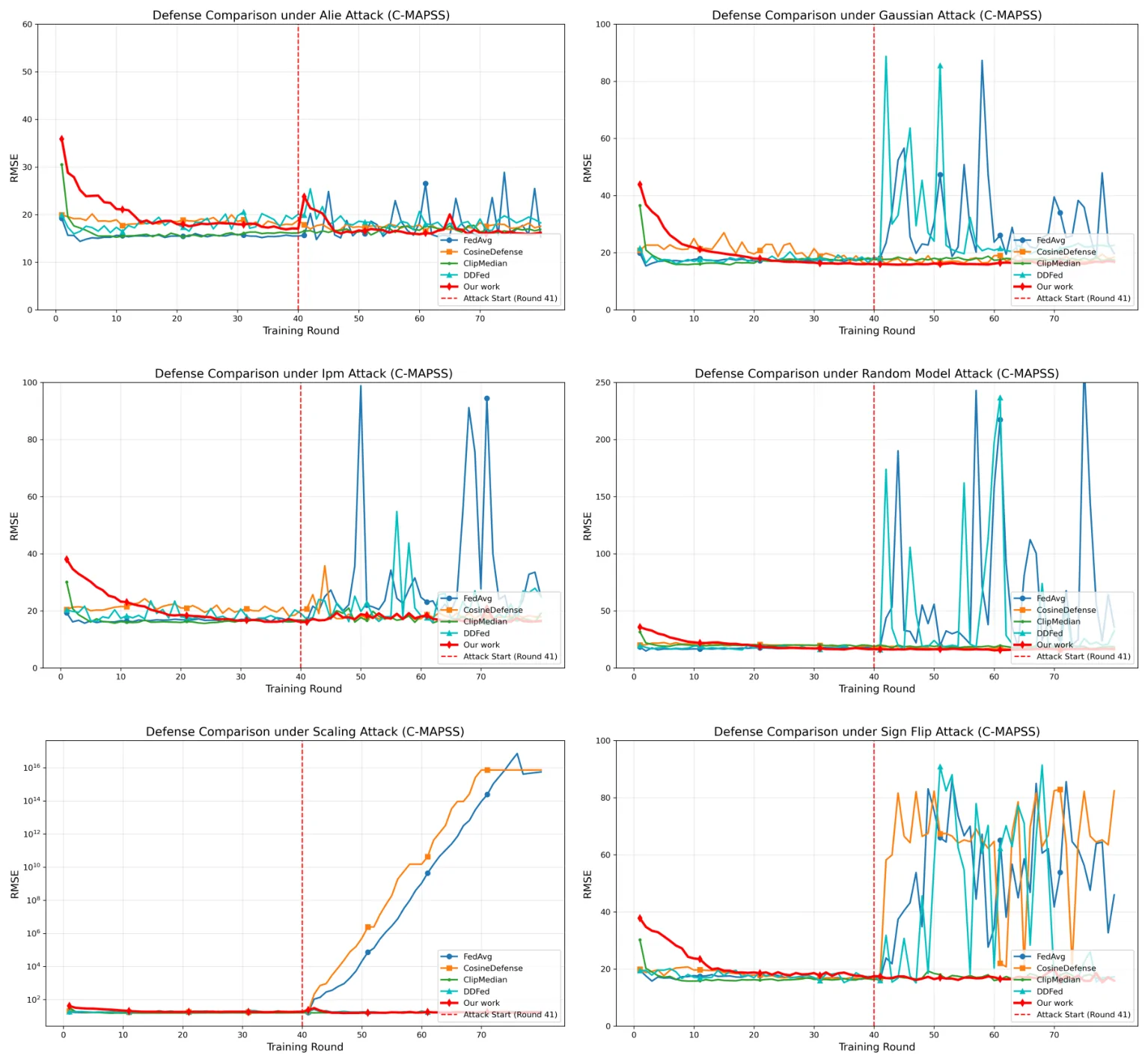
RMSE curves of different defense methods in various attack scenarios on the C-MAPSS dataset.

**Figure 6:**
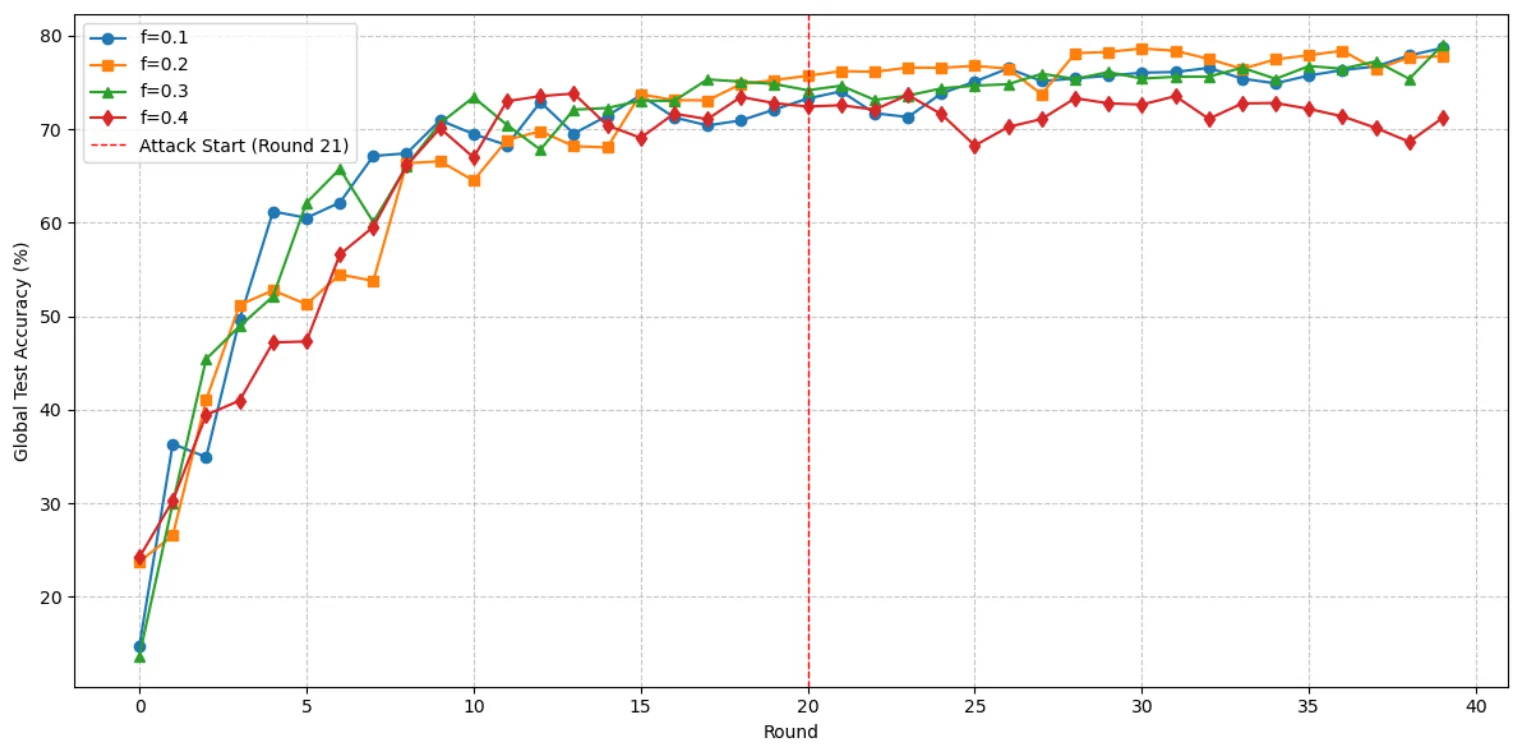
Impact of the malicious client ratio f on the global accuracy of the proposed method under scaling attack on Fashion-MNIST (attack from round 21).

Regarding the federated learning configuration, the total number of clients was K=20 for Fashion-MNIST, K=15 for MVTec AD, and K=10 for C-MAPSS. All clients participated in every communication round (full participation). The proportion of malicious clients was f=0.2 in the default configuration, with sensitivity analysis conducted for f∈{0.1,0.2,0.3,0.4}. For the main experiments shown in Figure 3, Figure 4 and Figure 5, attacks were injected starting from round $T_{\mathrm{attack}}=0.5\cdot T$, i.e., round 40 out of 80 for Fashion-MNIST/C-MAPSS and round 20 out of 50 for MVTec AD, ensuring that benign models had reached reasonable performance before attack onset. For the malicious-client-ratio experiment in Figure 6, a separate 40-round Fashion-MNIST experiment was conducted, with the attack injected from round 21.

To comprehensively evaluate robustness against different types of poisoning attacks, the experiments included representative malicious-update methods such as ALIE, Gaussian, IPM, Random Model, scaling, and Sign Flip attacks [29,30,31,32]. ALIE aims to construct malicious updates whose statistical distribution is close to that of normal clients to reduce the probability of detection; Gaussian attack increases the randomness of malicious updates by injecting random Gaussian perturbations; IPM attack uses inner-product manipulation to impose targeted interference on the global model; Random Model attack directly submits randomized model updates to the server; scaling attack amplifies the magnitude of local updates to strengthen malicious influence; and Sign Flip attack disrupts the normal convergence of the global model by reversing the gradient direction. These attack scenarios can comprehensively assess the proposed method’s ability to identify and suppress different forms of model poisoning.

For comparison, FedAvg, DualDefense, Cosine Defense, and Clipping Median were selected as baseline methods [5,28,33,34]. These methods represent no-defense aggregation, similarity-based defense, statistic-based robust aggregation, and joint defense that balances privacy protection with poisoning defense, respectively, making them highly representative. Comparing against these methods can more intuitively demonstrate the proposed scheme’s overall advantages in stability, attack resistance, and training efficiency.

For evaluation metrics, Fashion-MNIST mainly uses global test accuracy as the core metric, supplemented by convergence trends under attack, the number of rounds required for recovery after attack, and per-round training time; MVTec AD mainly uses image-level AUROC as the core metric; and C-MAPSS mainly uses RMSE or an equivalent regression-error metric to assess prediction performance, together with per-round training time to evaluate engineering deployability. By combining performance metrics with time overhead, the balance between theoretical security and practical efficiency of the proposed scheme can be evaluated comprehensively.

### 6.2. Experimental Results

As shown in Figure 3, Figure 4 and Figure 5, the performance of the proposed method was evaluated in different attack scenarios by tracking changes in model performance. Overall, on all three datasets, the proposed method exhibits good stability and robustness, maintaining relatively smooth performance curves after attack injection, whereas FedAvg shows obvious degradation under most attacks, indicating that direct averaging is difficult to resist poisoning updates when malicious clients participate. Meanwhile, Cosine Defense, Clipping Median, and DualDefense mitigate the performance loss caused by attacks to some extent, but their performance still fluctuates with different attack types, and defense effectiveness remains insufficiently stable under strong attacks.

On Fashion-MNIST, as shown in Figure 3, all methods converge quickly to a high test accuracy before attacks begin. After the attack starts, FedAvg suffers noticeable accuracy degradation under Gaussian, IPM, scaling, and Sign Flip attacks, and the degradation is particularly severe under Sign Flip attack, where accuracy drops rapidly to a low level. In contrast, the proposed method maintains a relatively smooth convergence trend under all attack types and preserves a high final accuracy, indicating that it can effectively suppress the damage caused by abnormal updates to the global model. Cosine Defense and DualDefense retain some performance in certain attack scenarios, but fluctuations remain visible after sustained attack, showing that their robustness is still affected by attack type. Clipping Median is relatively stable in some scenarios, but overall it is still slightly inferior to the proposed method.

On MVTec AD, as shown in Figure 4, all methods can achieve high AUROC levels before attacks begin. After attack injection, however, FedAvg exhibits the most severe performance degradation. In some attack scenarios, its AUROC rapidly falls to a level close to random discrimination, which demonstrates that it is highly sensitive to malicious updates in industrial anomaly-detection tasks. Cosine Defense, Clipping Median, and DualDefense can maintain favorable detection performance under certain attacks, yet their overall curves still show noticeable fluctuations. Under Gaussian, Random Model, and Sign Flip attacks, their final AUROC values suffer from varying degrees of loss compared with the pre-attack level. In contrast, the proposed method maintains higher and smoother AUROC curves across all tested attack scenarios, indicating a stronger ability to preserve the discriminative capability of the global model with malicious updates by more effectively identifying and suppressing malicious updates in industrial anomaly detection.

On C-MAPSS, as shown in Figure 5, the RMSE curve more directly reflects the stability of each method in the remaining-useful-life prediction task. The experimental results show that FedAvg is highly vulnerable under multiple attacks, especially under scaling attack, where RMSE diverges rapidly, indicating that it cannot cope with magnitude-amplification malicious updates. Under Gaussian, IPM, Random Model, and Sign Flip attacks, FedAvg also exhibits obvious error oscillations, demonstrating the lack of effective constraints on anomalous parameter updates. Although Cosine Defense, Clipping Median, and DualDefense can maintain lower error in some scenarios, they still fluctuate to varying degrees under strong attacks. In contrast, the proposed method maintains a relatively stable RMSE level under all six attacks and does not show obvious divergence after attack injection, indicating that the designed joint-defense mechanism can better guarantee training stability and prediction accuracy in time-series regression tasks.

As shown in Table 1, from the perspective of computation time, the proposed method introduces additional overhead compared with FedAvg because ciphertext-domain anomaly detection and multi-layer screening were performed during each communication round. The measured per-round training time is higher than FedAvg on Fashion-MNIST and MVTec AD, while the difference on C-MAPSS remains relatively small. These results indicate that the proposed defense introduces a measurable computational cost for privacy-preserving robust training. However, the current experiments mainly report end-to-end per-round execution time and do not separately quantify encryption latency, ciphertext expansion, communication volume, memory consumption, or scalability with the number of clients. Therefore, the present results should be regarded as an initial efficiency evaluation rather than a complete deployment-level overhead assessment.

### 6.3. Supplementary Evaluations

The foregoing experiments systematically validated the overall performance and poisoning defense capability of the proposed hierarchical secure federated learning framework across classification, anomaly detection, and regression tasks in non-IID industrial scenarios. To further examine the contribution of individual defense components and the robustness of the proposed method with different malicious-client ratios and hyperparameter settings, this section presents three complementary analyses: component ablation, malicious-client-ratio analysis, and hyperparameter sensitivity analysis.

To quantify the individual contribution of each defense component, we conducted comprehensive ablation experiments on Fashion-MNIST with three representative attack types (Gaussian, scaling, and Sign Flip). Table 2 reports the final accuracy after disabling one component at a time while keeping all others active.

Several observations emerge from Table 2. First, multi-layer detection is the most critical component: removing it causes the largest accuracy drop across all attack types (2.3–5.7 percentage points), confirming that single-layer similarity scoring is insufficient against sophisticated attacks. Second, update clipping is essential for defending against scaling attacks: its removal causes a 6.3-point drop under scaling attack but only modest degradation under other attacks, validating its targeted role in constraining magnitude-based attacks. Third, majority voting is most important under Sign Flip attack (3.8-point drop) because directional-manipulation attacks exploit individual scoring inconsistency that voting can aggregate away. Finally, the hierarchical DP budget and historical consistency terms provide moderate but consistent improvements (0.7–1.2 and 0.5–1.6 points respectively), justifying their inclusion despite their smaller individual contributions.

To investigate how the malicious client ratio affects the defense performance of the proposed method, we present in Figure 6 the variation in global accuracy with different malicious-client ratios *f* on the Fashion-MNIST dataset. When *f* = 0.1, 0.2, and 0.3, the accuracy curves exhibit similar convergence trends and remain within a relatively narrow range. When the malicious-client ratio increases to *f* = 0.4, the global accuracy shows a slight decline, which indicates that the increasing proportion of malicious updates gradually weakens the effectiveness of anomaly detection and benign-client selection. Nevertheless, the extent of such performance degradation remains relatively limited, indicating that the proposed method can still mitigate the impact of malicious clients to a certain degree at higher malicious client ratios.

To further verify the robustness of the proposed method under different parameter configurations, we conduct a hyperparameter sensitivity analysis, with the detailed results summarized in Table 3. For each parameter, we fix the other two at their default values (α=0.65,β=1.0,C=0.2) and vary the parameter of interest over a reasonable range. The results show that performance is stable within α∈[0.55,0.75], β∈[0.5,2.0], and C∈[0.1,0.4], with accuracy variations bounded within 1.5 percentage points. This insensitivity to precise parameter tuning supports the practical deployability of the method.

## 7. Conclusions and Future Work

This paper addresses the problems of data dispersion, privacy leakage, and poisoning attacks in cross-factory collaborative modeling for intelligent manufacturing and proposes a hierarchical privacy protection and poisoning-robust defense framework for industrial federated learning. By combining hierarchical privacy-budget allocation, multi-layer multi-feature anomaly detection, and ciphertext collaborative filtering, the proposed method achieves both privacy protection and robust training without sharing raw data and has been validated on Fashion-MNIST, MVTec AD, and C-MAPSS. The experimental results show that the proposed method maintains favorable performance stability under multiple poisoning attacks, exhibits strong applicability to intelligent-manufacturing tasks such as industrial anomaly detection and remaining-useful-life prediction, and keeps additional computational overhead generally controllable, demonstrating practical deployment value.

Future work will focus on further improving the generality, privacy assurance, and practical deployability of the proposed framework. First, more comprehensive comparisons with recent state-of-the-art robust federated-learning and secure-aggregation methods will be conducted with unified client partitions, attack configurations, and computational settings to further evaluate the relative advantages of the proposed approach. Second, the privacy protection mechanism will be further validated through quantitative experiments, including membership inference, gradient reconstruction, and feedback-based inference attacks, together with tighter privacy accounting and privacy–utility analysis under different privacy budgets. Third, more comprehensive adversarial evaluations will be considered, including adaptive attackers, coordinated malicious clients, targeted backdoor attacks, stronger non-IID heterogeneity, dynamic client participation, and varying data distributions. In addition, the scalability of CKKS-based computation will be systematically investigated in terms of communication overhead, ciphertext expansion, memory consumption, client scale, and model dimension, while lightweight encryption and communication-efficient strategies will be explored to improve suitability for resource-constrained industrial edge devices. Finally, future studies will further evaluate the framework using real industrial data and long-term online learning scenarios involving device heterogeneity, distribution drift, and multimodal sensing, thereby providing a more comprehensive assessment of its applicability to practical IIoT environments.

## Figures and Tables

**Table 1:** Comparison of per-round training time for different defense methods on each dataset.

Method	Fashion-MNIST	MVTec AD	C-MAPSS
	(s/Round)	(s/Round)	(s/Round)
FedAvg	21.77	54.22	10.17
Cosine Defense	22.24	55.13	10.67
Clipping Median	21.75	54.98	10.59
DualDefense	25.33	60.78	10.51
**Ours**	25.57	61.34	10.60

**Table 2:** Ablation study on Fashion-MNIST (*K* = 20, *f* = 0.2). Each row removes one component from the full method. The results are final accuracy (mean ± std over 5 runs).

Variant	Gaussian	Scaling	Sign Flip
Full method (Ours)	87.5 ± 0.8	86.1 ± 1.4	84.3 ± 1.8
–Hierarchical DP budget	86.8 ± 1.0	85.4 ± 1.6	83.5 ± 2.0
–Multi-layer detection	85.2 ± 1.5	83.1 ± 2.2	78.6 ± 3.2
–Historical consistency	86.9 ± 0.9	84.5 ± 1.8	83.7 ± 1.9
–Majority voting	85.8 ± 1.3	84.2 ± 1.9	80.5 ± 2.8
–Update clipping	86.3 ± 1.0	79.8 ± 3.5	83.0 ± 2.2

**Table 3:** Hyperparameter sensitivity on Fashion-MNIST (Gaussian attack, *f* = 0.2). Default values underlined.

Parameter	Final Accuracy (%)			
α	0.50: 86.1	0.60: 87.0	0.65: 87.5	0.75: 87.2
β	0.5: 86.8	1.0: 87.5	1.5: 87.3	2.0: 86.9
*C*	0.1: 86.3	0.2: 87.5	0.3: 87.1	0.4: 86.6

## Data Availability

The datasets used in this study are all publicly available. The Fashion-MNIST dataset is available at https://github.com/zalandoresearch/fashion-mnist (accessed on 7 May 2026). The MVTec AD dataset is available at https://www.mvtec.com/company/research/datasets/mvtec-ad (accessed on 14 May 2026). The C-MAPSS dataset is available at https://www.kaggle.com/datasets/palbha/cmapss-jet-engine-simulated-data (accessed on 26 May 2026). The implementation source code, client-partition configurations, attack-generation scripts, cryptographic parameter settings, and evaluation scripts are currently retained by the authors and are not publicly released at this stage due to intellectual property constraints and confidentiality agreements of the associated research project. To guarantee the reproducibility of this work, all critical experimental details have been documented in the manuscript. For additional technical inquiries, please correspond with the corresponding author.

## Abbreviations

L	Set of key layers involved in multi-layer anomaly detection
$S_{l}$	Sensitivity of layer *l*
$\varepsilon_{l}$	Privacy budget allocated to layer *l*
$\delta_{l}$	Failure probability parameter for layer *l*
${\tilde{h}}_{l}$	Perturbed representation at layer *l*
$\sigma_{l}$	Standard deviation of Gaussian noise at layer *l*
β	Control parameter for hierarchical privacy-budget allocation
$A_{k}^{\left(t\right)}$	Composite anomaly score of client *k* in round *t*
$\tau^{\left(t\right)}$	Dynamic screening threshold in round *t*
α	Weight balancing directional consistency and scale stability
$D_{k}^{\left(t\right)}$	Directional consistency term of client *k*
$S_{k}^{\left(t\right)}$	Scale stability term of client *k*
${\hat{B}}^{\left(t\right)}$	Candidate benign set returned by clients in round *t*
$B^{\left(t\right)}$	Final benign set obtained through majority voting in round *t*
$V_{i}^{\left(t\right)}$	Number of votes indicating client *i* is benign in round *t*
*C*	Clipping threshold
η	Numerical stability term
*f*	Proportion of malicious clients

## References

[1] Leng J., Li R., Xie J., Zhou X., Li X., Liu Q., Chen X., Shen W., Wang L. (2025). Federated learning-empowered smart manufacturing and product lifecycle management: A review. Adv. Eng. Inform..

[2] Farahani B., Monsefi A.K. (2023). Smart and collaborative industrial IoT: A federated learning and data space approach. Digit. Commun. Netw..

[3] Kuo T., Yang H. (2024). Federated learning on distributed and encrypted data for smart manufacturing. J. Comput. Inf. Sci. Eng..

[4] Boobalan P., Ramu S.P., Pham Q.V., Dev K., Pandya S., Maddikunta P.K.R., Gadekallu T.R., Huynh-The T. (2022). Fusion of federated learning and industrial Internet of Things: A survey. Comput. Netw..

[5] McMahan B., Moore E., Ramage D., Hampson S., y Arcas B.A. Communication-efficient learning of deep networks from decentralized data. Proceedings of the Artificial Intelligence and Statistics.

[6] Syed M.A.B., Rhaman Q., Sushil S. (2023). Federated learning in manufacturing: A systematic review and pathway to Industry 5.0. Proceedings of the 2023 5th International Conference on Sustainable Technologies for Industry 5.0 (STI), Dhaka, Bangladesh, 9–10 December 2023.

[7] Ge L., Li H., Wang X., Wang Z. (2023). A review of secure federated learning: Privacy leakage threats, protection technologies, challenges and future directions. Neurocomputing.

[8] Wei W., Liu L., Wu Y., Su G., Iyengar A. (2021). Gradient-leakage resilient federated learning. Proceedings of the 2021 IEEE 41st International Conference on Distributed Computing Systems (ICDCS), Virtual, 7–10 July 2021.

[9] Bai L., Hu H., Ye Q., Li H., Wang L., Xu J. (2024). Membership inference attacks and defenses in federated learning: A survey. ACM Comput. Surv..

[10] Xia G., Chen J., Yu C., Ma J. (2023). Poisoning attacks in federated learning: A survey. IEEE Access.

[11] Chen C., Liao T., Deng X., Wu Z., Huang S., Zheng Z. (2025). Advances in robust federated learning: A survey with heterogeneity considerations. IEEE Trans. Big Data.

[12] Han Q., Lu S., Wang W., Qu H., Li J., Gao Y. (2024). Privacy preserving and secure robust federated learning: A survey. Concurr. Comput. Pract. Exp..

[13] Akhtarshenas A., Vahedifar M.A., Ayoobi N., Maham B., Alizadeh T., Ebrahimi S., López-Pérez D. (2024). Federated learning: A cutting-edge survey of the latest advancements and applications. Comput. Commun..

[14] Guembe B., Misra S., Azeta A. (2024). Privacy issues, attacks, countermeasures and open problems in federated learning: A survey. Appl. Artif. Intell..

[15] Chouhan A., Purushothama B.R. (2023). A survey on secure aggregation for privacy-preserving federated learning. International Conference on Advancements in Smart Computing and Information Security.

[16] Cooray L., Sendanayake J., Vithanaarachchi P., Priyadarshana Y.H.P.P. (2025). Deep federated learning: A systematic review of methods, applications, and challenges. Front. Comput. Sci..

[17] Shenoy D., Bhat R., Krishna Prakasha K. (2025). Exploring privacy mechanisms and metrics in federated learning. Artif. Intell. Rev..

[18] Alahmari S., Alghamdi I. (2025). A comprehensive survey on energy-efficient and privacy-preserving federated learning for edge intelligence and IoT. Results Eng..

[19] Uddin M.P., Xiang Y., Hasan M., Bai J., Zhao Y., Gao L. (2025). A systematic literature review of robust federated learning: Issues, solutions, and future research directions. ACM Comput. Surv..

[20] Khan N., Nisar S., Khan M.A., Rehman Y.A.U., Noor F., Barb G. (2025). Optimizing federated learning with aggregation strategies: A comprehensive survey. IEEE Open J. Comput. Soc..

[21] Zhang J., Cooper C., Gao R.X. (2022). Federated learning for privacy-preserving collaboration in smart manufacturing. Global Conference on Sustainable Manufacturing.

[22] Deng T., Li Y., Liu X., Wang L. (2023). Federated learning-based collaborative manufacturing for complex parts. J. Intell. Manuf..

[23] Shen J., Zhao Y., Huang S., Ren Y. (2024). Secure and flexible privacy-preserving federated learning based on multi-key fully homomorphic encryption. Electronics.

[24] Guo Y., Li L., Zheng Z., Yun H., Zhang R., Chang X., Gao Z. (2024). Efficient and privacy-preserving federated learning based on full homomorphic encryption. arXiv.

[25] Pan Y., Chao Z., He W., Jing Y., Hongjia L., Liming W. (2024). FedSHE: Privacy preserving and efficient federated learning with adaptive segmented CKKS homomorphic encryption. Cybersecurity.

[26] Abadi M., Chu A., Goodfellow I., McMahan H.B., Mironov I., Talwar K., Zhang L. Deep learning with differential privacy. Proceedings of the 2016 ACM SIGSAC Conference on Computer and Communications Security.

[27] Blanchard P., El Mhamdi E.M., Guerraoui R., Stainer J. Machine learning with adversaries: Byzantine tolerant gradient descent. Proceedings of the 31st International Conference on Neural Information Processing Systems, NIPS’17.

[28] Xu R., Gao S., Li C., Joshi J., Li J. (2024). Dual defense: Enhancing privacy and mitigating poisoning attacks in federated learning. Adv. Neural Inf. Process. Syst..

[29] Baruch G., Baruch M., Goldberg Y. (2019). A Little Is Enough: Circumventing Defenses For Distributed Learning. Advances in Neural Information Processing Systems.

[30] Xie C., Koyejo O., Gupta I. Fall of empires: Breaking byzantine-tolerant SGD by inner product manipulation. Proceedings of the 35th Uncertainty in Artificial Intelligence Conference, PMLR.

[31] Bagdasaryan E., Veit A., Hua Y., Estrin D., Shmatikov V. How to backdoor federated learning. Proceedings of the Twenty Third International Conference on Artificial Intelligence and Statistics, PMLR.

[32] Colosimo F., De Rango F. (2024). Dynamic gradient filtering in federated learning with Byzantine failure robustness. Future Gener. Comput. Syst..

[33] Yaldiz D.N., Zhang T., Avestimehr S. (2023). Secure federated learning against model poisoning attacks via client filtering. arXiv.

[34] Yin D., Chen Y., Kannan R., Bartlett P. Byzantine-robust distributed learning: Towards optimal statistical rates. Proceedings of the 35th International Conference on Machine Learning, PMLR.

